# A Colorimetric Method for Monitoring Tryptic Digestion Prior to Shotgun Proteomics

**DOI:** 10.1155/2014/125482

**Published:** 2014-02-10

**Authors:** Richard I. Somiari, Kutralanathan Renganathan, Stephen Russell, Steven Wolfe, Florentina Mayko, Stella B. Somiari

**Affiliations:** ^1^ITSI Biosciences, 633 Napoleon Street, Johnstown, PA 15901, USA; ^2^Windber Research Institute, 620 Seventh Street, Windber, PA 15963, USA

## Abstract

Tryptic digestion is an important preanalytical step in shotgun proteomics because inadequate or excessive digestion can result in a failed or incomplete experiment. Unfortunately, this step is not routinely monitored before mass spectrometry because methods available for protein digestion monitoring either are time/sample consuming or require expensive equipment. To determine if a colorimetric method (ProDM Kit) can be used to identify the extent of tryptic digestion that yields the best proteomics outcome, plasma and serum digested for 8 h and 24 h were screened with ProDM, Bioanalyzer, and LC/MS/MS, and the effect of digestion on the number of proteins identified and sequence coverage was compared. About 6% and 16% less proteins were identified when >50% of proteins were digested in plasma and serum, respectively, compared to when ~46% of proteins were digested. Average sequence coverage for albumin, haptoglobin, and serotransferrin after 2 h, 8 h, and 24 h digestion was 52%, 45%, and 45% for serum and 54%, 47%, and 42% for plasma, respectively. This paper reiterates the importance of optimizing the tryptic digestion step and demonstrates the extent to which ProDM can be used to monitor and standardize protein digestion to achieve better proteomics outcomes.

## 1. Introduction

Proteomics has advanced significantly over the past decade [1]. This rapidly evolving technology is now routinely applied in many laboratories for protein expression profiling, biomarker discovery/validation, posttranslational modification mapping, and complex disease research [2]. Mass spectrometry remains the predominant technology driving proteomics and this technology continues to evolve. The depth of sampling and sensitivity and the scan speed of mass spectrometers have improved tremendously. But critical sample preparation steps which could impact the success of proteomics experiments, for example, the digestion of proteins into peptides prior to the mass spectrometry step, have not improved or received the kind of attention that instrumentation receives.

The variant of proteomics termed shotgun proteomics, also known as bottom-up proteomics, is one of the most widely applied proteomic strategies which rely on the digestion of proteins into peptides prior to mass spectrometry. In shotgun proteomics, an enzyme such as trypsin is added to the protein sample and the mixture is incubated at 37°C for a specified time or overnight at ambient room temperature to digest proteins. Trypsin cleaves proteins at the C-terminus of Lysine (K) or Arginine (R) residues, except when either residue is followed by a Proline (P). The resulting peptides are analyzed by mass spectrometry and the mass-to-charge (*m*/*z*) ratio generated is used to establish protein identities by reference to an appropriate database. The protein digestion step is recognized as one of the single most important sample preparation steps that can directly affect the outcome of all proteomics experiments. Since the central objective of all shotgun proteomics is to identify all proteins present in the sample in a single run, it is desirable and very important to optimize all pre-analytical and sample preparation steps especially the protein digestion step, to ensure that there is run-to-run reproducibility and the maximum possible number of proteins in each sample will be identified in each run.

Zhang and Li (2004) reports that the type of protein, complexity of sample mixture, presence of surfactants, and impurities in the reaction mixture can affect the efficiency of tryptic digestion [3] and hence the outcome of the experiment. Considering that trypsin cleaves proteins at the C-terminus of Lysine or Arginine residues except when either residue is followed by a Proline, it is expected that the time required to sufficiently digest proteins with trypsin will vary amongst different protein types, sample complexity, and reaction condition. That means the addition of trypsin (or any selected enzyme) to a protein mixture and incubation of the reaction mixture for a specified time period is not a guarantee that the enzyme will sufficiently digest all classes of protein(s) present within the time allowed for optimal proteomics outcome. In fact the study of Klammer and MacCoss published in 2006 clearly demonstrated that the number of unique proteins identified in plasma is primarily determined by the quality and completeness of the tryptic digestion step [4], and Karuso et al. [5] recommend that proteins should be optimally digested prior to mass spectrometry, to avoid the wastage of valuable instrument time and generation of results that will be difficult to interpret. But most laboratories add trypsin to samples and incubate for a time period with the hope that the digestion obtained will be sufficient for optimal mass spectrometry. No attempt is typically made to determine if the digestion is sufficient before the mass spectrometry step. Experience in our laboratory indicate that the same batch of trypsin often digests different protein samples to different degrees within a specified time due to sample complexity, sequence differences, and specific activity of the enzyme. Considering that under the same conditions, two different protein samples exposed to trypsin may not be digested to the same extent, using a fixed digestion time will not result in optimal digestion of all types of proteins. Therefore, to objectively compare mass spectrometry results, the tryptic digestion step must be standardized and a better method for standardization should be based on the “extent” rather than “duration” of digestion.

It is reported that routine monitoring of tryptic digestion for understanding extent or percentage of protein digestion is not performed in most laboratories partly because the methods available to monitor protein digestion including HPLC, circular dichroism, SDS-PAGE, mass spectrometry, and the use of fluorescent dyes are both time and sample consuming, and expensive to perform on a regular basis, or require special and expensive equipment [5, 6]. Methods for protein digestion monitoring that rely on the use of a mass spectrometer is unattractive and challenging because of the cost and time for the mass spectrometry analysis, database search to identify the protein sequence and deducing sample independent metrics that can be used to monitor the completion of trypsin digestion. Additionally, because of scanty scientific literature on the subject, many proteomics scientists are unaware of the significance of insufficient and excessive digestion on the outcome of proteomics experiments, and many erroneously believe that the only requirement for a “good” proteomics result is access to a state-of-the-art high-end mass spectrometer.

We were motivated to conduct this study because our laboratory routinely performs shotgun proteomics of plasma and serum samples using label-free LC/MS/MS and iTRAQ technologies, and we observe differences in protein identification that were attributed to inconsistent trypsin digestion. Since reproducibility and maximum protein identification are essential in quantitative proteomics, we presumed that using a method that is independent of mass spectrometry to standardize the tryptic digestion step will improve reproducibility and outcome. This paper describes the evaluation of a novel protein digestion monitoring (ProDM) kit, as a method for monitoring trypsin digestion prior to mass spectrometry. The results obtained from our experiments demonstrate that the number of proteins identified and the protein sequence coverage are affected by the extent of protein digestion, and ProDM can be used to precisely determine the extent of protein digestion before the mass spectrometry step.

## 2. Materials and Methods

The ammonium bicarbonate, acetonitrile, and Trifluoroethanol were purchased from Fisher Scientific, Pittsburgh, PA, iodoacetamide (IAA) was purchased from GE Healthcare Biosciences, Piscataway, NJ, formic acid from Sigma, St. Louis, MO, and *β*-mercaptoethanol was purchased from Amresco, Solon, OH. The Agilent Protein 80 chip kit was purchased from Agilent Technologies, Santa Clara, CA. The Proteomics Grade Plasma (PGPT) and Serum (PGST) tubes for collection of blood, protein digestion monitoring Kit (ProDM), and Total Protein Assay Reagent (ToPA) were supplied by ITSI-Biosciences, Johnstown, PA, and PicoFrit C18 nanospray column was purchased from New Objective, Woburn, MA.

### 2.1. Blood Collection and Sample Preparation

Blood was collected from a 45-year-old male donor using the PGPT and PGST tubes to eliminate ex vivo changes that could skew the data. Plasma and serum were isolated according to standard plasma and serum isolation protocols, aliquoted in 1 mL amounts to avoid repeated freeze/thaw of the same aliquot, and stored at −80°C until used. Prior to use, samples were thawed on ice and the total protein content was determined for all samples using the ToPA Kit. All experiments were performed in duplicate and all results presented are averages of two separate readings.

### 2.2. Digestion of Proteins with Trypsin

Plasma and serum proteins were digested with trypsin in a buffer system containing Trifluoroethanol (TFE). Briefly, 50 *μ*L of plasma or serum was added to a clean microfuge tube containing 50 *μ*L of TFE, and the mixture vortexed briefly. The sample was reduced with DTT (10 mM) and alkylated with IAA (20 mM). 400 *μ*L of HPLC grade water was added to dilute the TFE and prevent the interference from TFE. The pH was checked and appropriate amount of 1 M ammonium bicarbonate was added so that the final concentration of ammonium bicarbonate was 50 mM and the pH was above 8.0. Trypsin (5% w/w) was added and an aliquot representing “time zero” was removed and processed immediately. Subsequently, the reaction mixture was incubated for 8 h and 24 h at 37°C. At the end of the incubation, 5% formic acid (v/v) was added to stop the reaction. The time zero, 8 h, and 24 h samples were analyzed as described below.

### 2.3. Effect of Digestion Time on Protein Sequence Coverage

In a parallel experiment serum and plasma were digested as described above for 2 h, 8 h, and 24 h and the digested samples were analyzed by mass spectrometry. The sequence coverage of three model proteins, namely, albumin, haptoglobin, and serotransferrin was determined and compared to elucidate the effect of digestion extent on protein sequence coverage.

#### 2.3.1. Monitoring of Tryptic Digestion with ProDM

Plasma and serum samples were analyzed with the ProDM kit according to the manufacturers' protocol. Briefly, ProDM Kit contains *ready-to-use* reagents including (i) a Standard Buffer (Urea-Tris, Buffer pH 8.5), (ii) Reaction Buffer (Tris-Buffer, pH 8.5), (iii) Reaction Quencher (Buffered Phosphoric acid), and (iv) modified ToPA colorimetric Reagent (ITSI-Biosciences, Johnstown, PA). The samples collected at time zero and the samples collected after 8 h and 24 h digestion with trypsin were independently processed to determine the extent of protein digestion. 10 *μ*L of the reaction mixture was transferred, at zero time and after 8 h and 24 h incubation, to a fresh Eppendorf tube containing 2 *μ*L of Reaction Quencher. The mixture was vortexed briefly to mix, colorimetric reagent was added, and the absorbance was read at 595 nm within 1–3 min of adding the color reagent. The % protein digested (%PD) was calculated with an application  running in MS EXCEL. The application is based on the formula %PD = [(*A*_1(*T*0,*l*)_ − *A*_2(*Tx*,*l*)_)/*A*_1(*T*0,*l*)_]∗100, where %PD is the percentage of protein digested by the enzyme into peptides within a specific incubation time interval; *A*_1(*T*0,*l*)_ is the absorbance of the first aliquot at time zero; and *A*_2(*Tx*,*l*)_ is the absorbance of the second aliquot after 8 h or 24 h digestion.

#### 2.3.2. Monitoring of Tryptic Digestion with the Bioanalyzer Protein 80 chip

To screen the samples with the Agilent Bioanalyzer Protein 80 chip, 50 *μ*L each of (a) time zero, (b) 8 h digested, and (c) 24 h digested serum and plasma samples were transferred to fresh tubes, dried down in a Speedvac, and resuspended with 30 *μ*L of distilled and deionized (Milli-Q) water. Then, 4 *μ*L of the resuspended sample containing 3.4 *μ*g of total protein was transferred to a clean tube and 2 *μ*L of Agilent sample buffer supplemented with 3.5% v/v of *β*-mercaptoethanol was added. The samples and 6 *μ*L of protein ladder included in the Protein 80 kit were heated for 5 min at 95°C and diluted with 84 *μ*L of Milli-Q water before electrophoresis. For electrophoresis, the Agilent Protein 80 chip was primed according to the manufacturer's instructions and 6 *μ*L of all samples was loaded into separate sample wells and the chip was run on the Agilent Bioanalyzer 2100 using the Protein 80 Assay program.

### 2.4. Mass Spectrometry to Determine Effect of Digestion Time on the Number of Identified Proteins and Protein Sequence Coverage

Following trypsin digestion for 8 h and 24 h, the digestion mixtures were acidified with 5% (v/v) formic acid and dried down in a Speedvac. The dried sample was reconstituted in 2% acetonitrile/0.1% formic acid and loaded onto a PicoFrit C18 nanospray column using a Thermo Scientific Surveyor Autosampler operated in no waste injection mode [7]. Peptides were eluted from the column using a linear acetonitrile gradient from 2% to 40% over 60 minutes into a LTQ XL mass spectrometer (Thermo Scientific) via a nanospray source with the spray voltage set to 1.8 kV and the ion transfer capillary set at 180°C. A data-dependent Top 5 method was used where a full MS scan from *m*/*z* 400–1500 was followed by MS/MS scans on the five most abundant ions [7]. Protein identification and the number of missed cleavages were determined with the Proteome Discoverer 1.3 software as previously described [7].

Briefly, the raw data files were searched utilizing SEQUEST algorithm in Proteome Discoverer 1.3 (Thermo Scientific) against the most recent species-specific FASTA database for human downloaded from NCBI. Trypsin was the selected enzyme and we allowed for up to three missed cleavages per peptide. Carbamidomethyl Cysteine was used as a fixed modification. Precursor and fragment ion peaks were searched with a mass tolerance of 5000 ppm and 2 Da, respectively. Proteins were identified when unique peptides had X-correlation scores greater than 1.5, 2.0, and 2.5 for respective charge states of +1, +2, and +3 [7]. To test for optimal-specificity and nonspecific cleaving of proteins the database was searched with full and semispecificity setting for trypsin. Since, contaminating chymotrypsin activity may contribute to generation of nontryptic peptides a similar search was also conducted using chymotrypsin with full specificity. To identify other parameters that may be different as a result of different tryptic digestion times, we compared the *m*/*z* distribution and ion intensities in 8 h and 24 h samples by plotting 2D density maps consisting of time (*x*-axis) versus *m*/*z* (*y*-axis) versus relative abundance (*z*-axis) for both samples using Xcalibur 2.1 (Thermo Scientific). Proteome Discoverer 1.3 was utilized to plot the graph of *m*/*z* versus peptide length to demonstrate the distribution of charges in the digested plasma and serum samples.

## 3. Results and Discussion

The primary goal of this study was to determine if ProDM, a novel protein digestion monitoring kit, can be used to monitor the pre-analytical trypsin digestion step to identify the precise extent of protein digestion that gives the best protein identification and sequence coverage outcome. Since ProDM is a colorimetric method we also analyzed the samples with the Agilent Bioanalyzer Protein 80 chip, to benchmark and verify the ProDM data. To determine how the extent of digestion affects the outcome of the mass spectrometry data, we analyzed the digested samples by tandem mass spectrometry and compared (a) the number of proteins identified and (b) protein sequence coverage.

The ProDM analysis revealed that about 46% of plasma and serum proteins were digested after 8 h incubation, whereas after 24 h digestion, 56% of proteins were digested in plasma and 50% in serum (Table 1). This simple colorimetric method required 10 *μ*L of sample and less than 15 min to complete. Although the mechanism of action of ProDM has not been fully elucidated, preliminary studies indicate that the modified ToPA reagent used in the reaction binds to full length proteins and not peptides. The color at time zero (no digestion) when it is expected that there will be more full length proteins is consistently more intense (higher absorbance) than the color after tryptic digestion (lower absorbance) when fewer full length proteins are expected.

**Figure 1:**
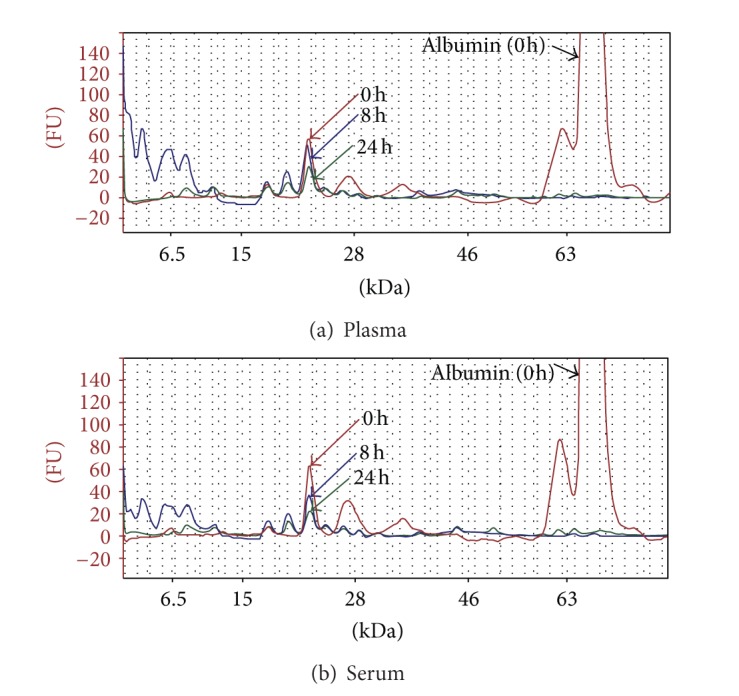
Agilent Bioanalyzer protein 80 electrophoregram of undigested and digested plasma (a) and serum (b). Although albumin is extensively digested within 8 h there are protein peaks still visible in the 24 h digested samples especially around the 20 kDa and 28 kDa range. Red line is 0 h, blue line is 8 h, and green line is 24 h.

This reduction in color intensity (and absorbance) correlates with the disappearance (or reduction) in peak height of higher molecular weight proteins as revealed by SDS-PAGE (results not presented) and Agilent Bioanalyzer data (Figure 1). Therefore, the data for % protein digested is the result of undigested and partially digested proteins in the samples at the time of sample collection. Thus, ProDM will provide information on the difference between time intervals and cannot differentiate between enzymatic and nonenzymatic digestions.

Analysis of aliquots of the digested samples with the Agilent BioAnalyzer Protein 80 chip revealed extensive digestion of abundant proteins, especially albumin (Figure 1). The Bioanalyzer also showed that there are still partially digested proteins particularly in the 20 kDa to 50 kDa region after 24 h incubation (Figure 1). The visible peaks around 6.5 kDa (Figure 1) in the 8 h samples are products of partial tryptic digestion of larger proteins. This assumption is plausible because these peaks are significantly lower in the 24 h digested samples (Figure 1). The Agilent Bioanalyzer data correlated with the ProDM results and provided a gel-based verification of the presence of undigested proteins after 8 h and 24 h digestion with trypsin. This means that ProDM, which gives the precise percentage of proteins digested, can be used to determine the extent of trypsin digestion that will give the best proteomics outcome.

The goal of all shotgun proteomics experiments is to identify as many proteins as possible with high confidence, and the more the percentage of sequence coverage the higher the confidence. To determine how the tryptic digestion step can affect the outcome of shotgun proteomics, we analyzed the samples digested for different extents and compared the number of proteins identified and sequence coverage of three proteins commonly found in plasma and serum. As shown in Table 1, a total of 991 and 733 peptides were sequenced in 8 h and 24 h digested plasma samples, whereas a total of 897 and 693 peptides were sequenced in 8 h and 24 h digested serum samples, respectively. The corresponding total number of unique proteins identified in plasma in the 8 h and 24 h samples was 125 and 118, and in serum this was 127 and 107, respectively (Table 1). This represents about 26% reduction in the number of peptides sequenced and 6% drop in the number of proteins identified in 24 h digested samples compared to 8 h digested samples, respectively. In serum 127 proteins were identified in 8 h digested samples and 107 were identified in 24 h digested samples (Table 1). This represents about 16% reduction in the number of proteins identified in 24 h digested samples compared to 8 h digested samples. The finding that longer digestion times can result in reduced number of identified proteins has been previously reported [6], and the 125 unique proteins we identified in plasma after 8 h tryptic digestion are comparable to the 150 proteins identified by Zimmerman et al. using a comparable LC/MS/MS equipment and approach [8], suggesting that our finding could be generalized to some extent. Since 24 h digestion apparently resulted in fewer number of identified proteins compared to 8 h, the ProDM data obtained in this experiment shows that ~46% digestion of plasma and serum yields greater numbers of identified proteins compared to 24 h digestion which resulted in ≥50% protein digestion.

**Figure 2:**
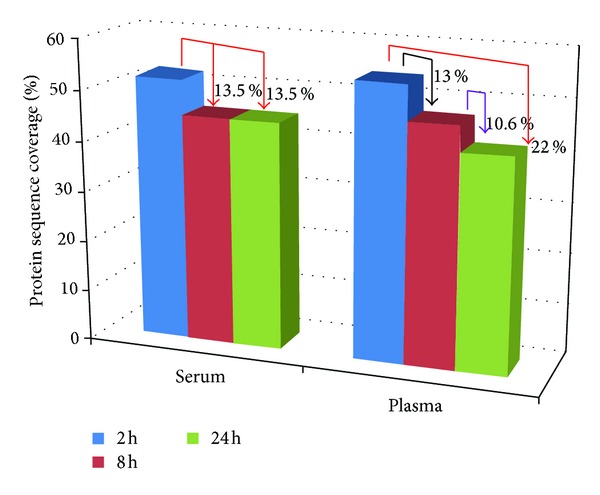
Average protein sequence coverage for albumin, haptoglobin, and serotransferrin in serum and plasma after tryptic digestion for 2 h, 8 h, and 24 h. In serum, the average sequence coverage in 8 h and 24 h digested sample was 13.5% lower than in 2 h, and in plasma the average coverage in 8 h was 13% lower than in 2 h, and average coverage in 24 h was 22% lower than in 2 h.

In a parallel experiment, we used albumin, haptoglobin, and serotransferrin as models to gain insights into the potential effect of the % of digestion on protein sequence coverage. These three proteins have different molecular weights, and together they account for over 70% of the total proteins in serum. As shown in Figure 2, the average sequence coverage for the three proteins in the 2 h, 8 h, and 24 h samples for Serum were 52%, 45%, and 45%, respectively, and for plasma were 54%, 47%, and 42%, respectively.

Specifically, in plasma, the sequence coverage in the 2 h samples ranged from about 47% (haptoglobin) to 61% (albumin) and the coverage in 24 h samples ranged from about 30% (haptoglobin) to 52% (albumin). In Serum, the coverage in the 2 h samples ranged from 32% (haptoglobin) to 70% (albumin), whereas the coverage in the 24 h samples ranged from 31% (haptoglobin) to 61% (albumin). It was interesting to observe that the average coverage in 2 h was consistently higher than the coverage in 24 h for the three proteins in serum and plasma. This finding is noteworthy because it suggests that excessive digestion may not only result in reduced number of the total proteins identified, but could also lead to lower confidence due to a reduced protein sequence coverage. If optimal sequence coverage is required, then ProDM could be used to determine the least percentage of digestion that gives the best coverage for the target protein. Additional benefits to finding and using the shortest digestion time that will give the best results include the ability to perform more experiments per day and savings on labor cost.

**Figure 3:**
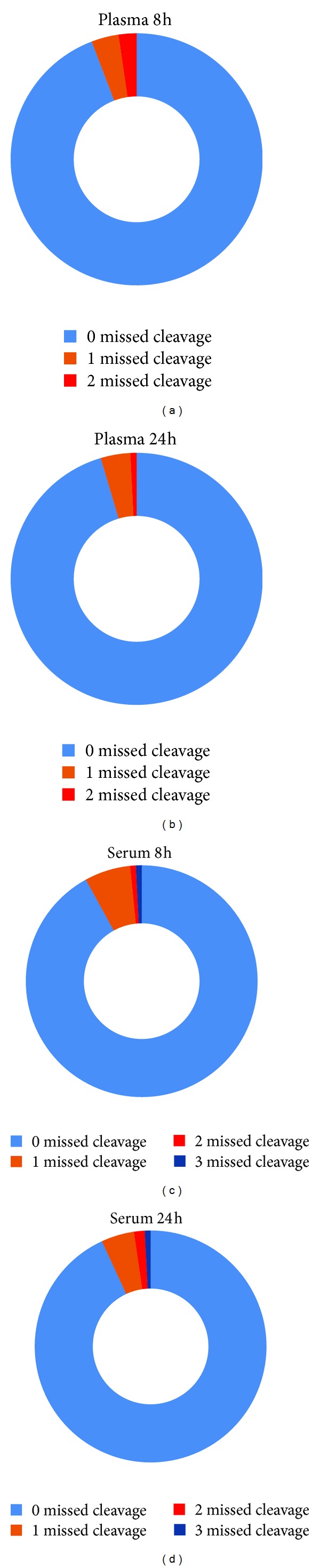
Number of missed cleavages in plasma and serum proteins digested for 8 h and 24 h with trypsin. Chart shows the percentage (%) of peptides with no missed cleavage (0) and peptides with one (1), two (2), and three (3) missed cleavages.

To determine why longer digestion times resulted in fewer number of proteins identified and less sequence coverage we postulated that the “number of missed cleavages in the 8 h and 24 h digested samples will be different.” We were interested in missed cleavages because it could provide insights into the specific activity of the enzyme as the digestion progressed. Interestingly, there was no dramatic difference in the number of missed cleavages between the 8 h and 24 h samples, except for “2 missed cleavages,” where for plasma 2.3% was detected in 8 h and 0.8% was detected in 24 h and for serum 0.8% was detected in 8 h and 1.5% was detected in 24 h (Figure 3).

**Figure 4:**
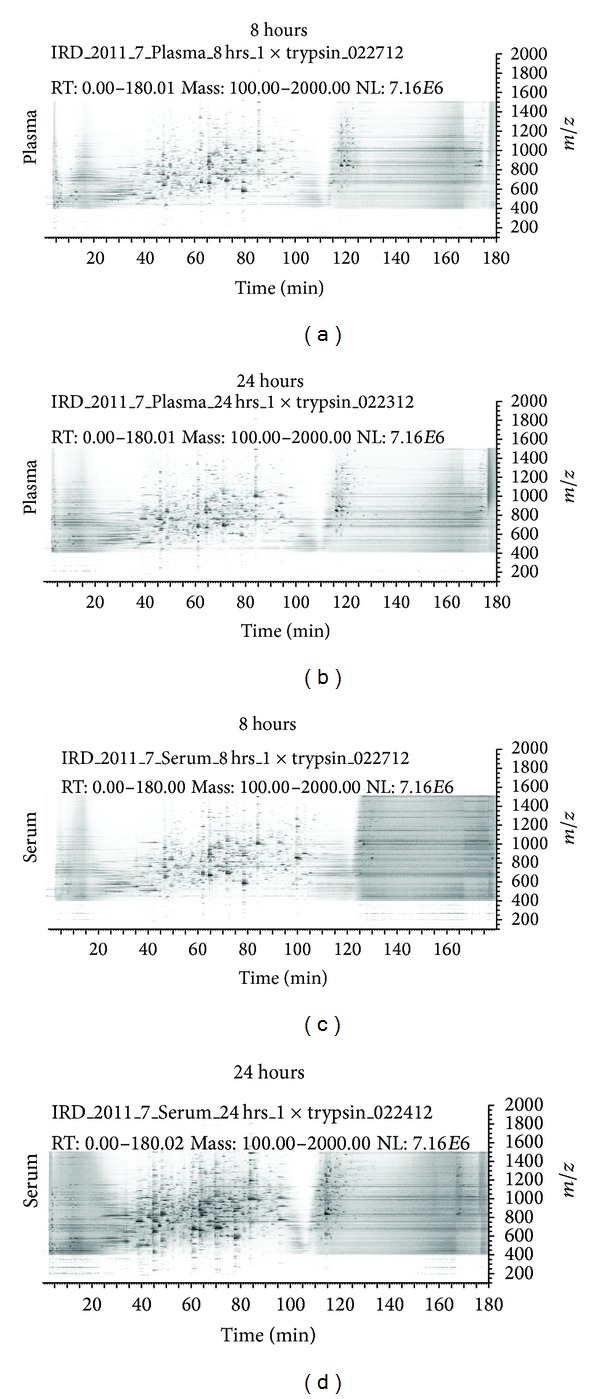
2D density maps consisting of time (*x*-axis) versus *m*/*z* (*y*-axis) versus relative abundance (*z*-axis) for serum and plasma samples digested for 8 h and 24 h. Only a subtle difference can be detected showing that there was a good run-run reproducibility.

The absence of a major difference in the number of “zero” missed cleavages between 8 h and 24 h digestion is remarkable and requires more study. To determine if this phenomenon is related to reproducibility between mass spectrometry runs, a plot consisting of time (*x*-axis) versus *m*/*z* (*y*-axis) versus relative abundance (*z*-axis) was used to compare the 8 h and 24 h digested samples.  Figure 4 shows only a subtle difference between 8 h and 24 h samples for plasma and serum indicating that there was good run-run reproducibility. Thus, the difference in the number of proteins identified is apparently not likely due to reproducibility.

The finding that 24 h digestion compared to 8 h digestion leads to a decrease in the number of proteins identified and % protein sequence coverage underscores the importance of optimizing and standardizing the enzymatic digestion step prior to mass spectrometry. One postulate is that extended digestion leads to poor identifications because of loss of enzyme specificity over time. If this is the case, then a search of the database using chymotrypsin or semitrypsin as the selected enzyme should produce a new set of identified proteins. Indeed, a database search with chymotrypsin using the raw MS/MS files identified a new set of proteins and resulted in a 12% increase in the coverage for albumin in the 24 h sample. This demonstrates the presence of more nontryptic peptides in the 24 h samples compared to 8 h samples. It is therefore likely that fewer proteins were identified in the 24 h samples probably because of the loss of enzyme specificity, which resulted in the production of non-/semitryptic peptides.

**Figure 5:**
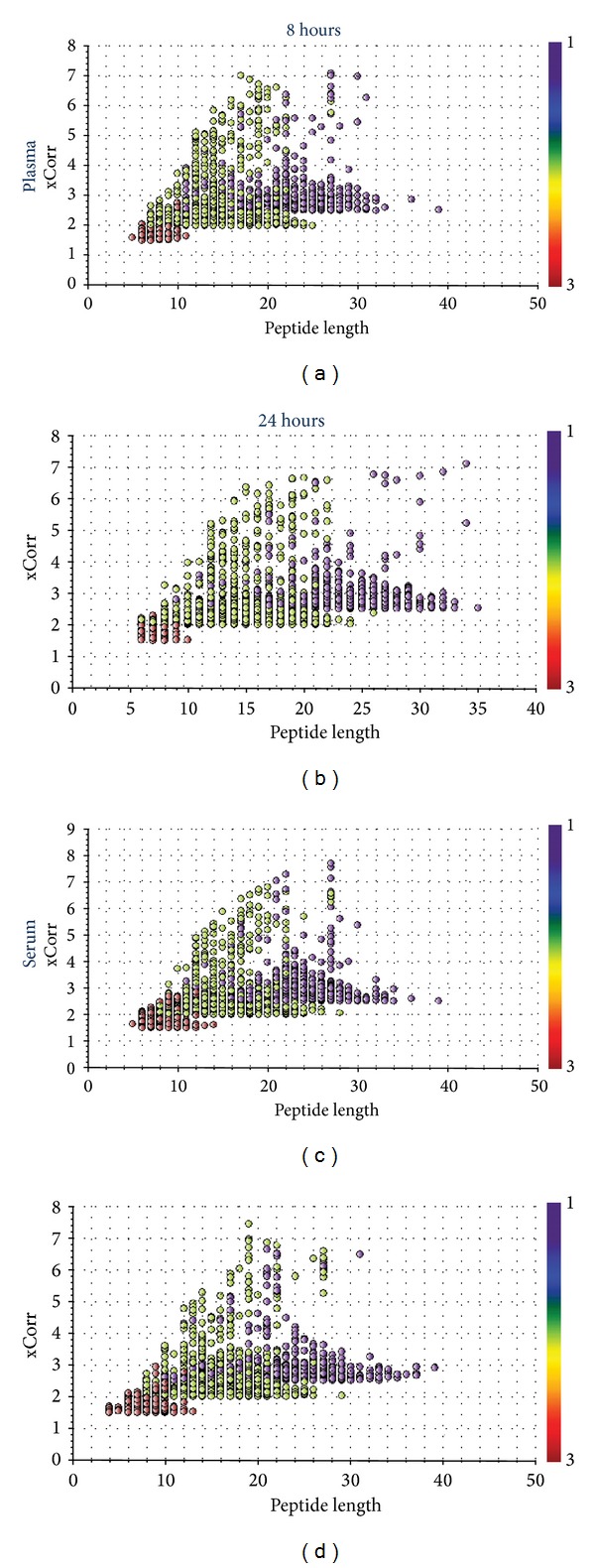
Plot of xCorr versus peptide length demonstrating the distribution of +1 to +3 charges in 8 h and 24 h digested serum and plasma samples. The +2 charges are more in the 8 h samples compared to 24 h.

The charge state of peptides in the 8 h and 24 h samples were compared because the presence of peptides with higher charges is an indication of the presence of longer peptides [9]. As shown in Table 2, 72% of all peptides identified in plasma and 86% of all peptides identified in serum in the 8 h samples had +2 charge states. The 24 h samples contained a higher number of +3 charges indicating that there were more longer peptides compared to the 8 h samples. The graphical illustration of the distribution of charges obtained by plotting the xCorr versus peptide length clearly shows the presence of more +2 charge states in the 8 h samples compared to 24 h (Figure 5).

The finding that 24 h samples contained longer peptides is interesting since they should ideally have fewer undigested peptides due to enzymatic activity. It is therefore likely that overall, while there is likely a reduction in the total enzyme activity and a loss of specific activity in 24 h samples, there are other random or nonspecific activities at play that result in the presence of relatively more peptides that are longer and nontryptic peptides in 24 h samples. Since protein identification by mass spectrometry is optimal for +2 charge state peptides compared to +3 [9], the presence of longer peptides in 24 h samples could partly explain why fewer number of proteins were identified in the 24 h digested samples under our experimental conditions.

The tryptic digestion step is a critical and the most time consuming sample preparation step [10]. Although many studies including that of Klammer and MacCoss [4] demonstrate that the trypsin digestion step is a limiting factor in the efficiency of protein identification by mass spectrometry, many researchers still do not optimize or standardize this process, and this raises the question of how the digestion efficiency is evaluated and compared within and between laboratories. The amino acid sequence coverage (SQ%) has been reported as a measure that can be used to determine both the completeness of the protein digestion and the detection efficiency of the various tryptic peptides [10] and digestion rate [11]. But the use of SQ% might be misleading because different mass spectrometers and different search parameters may reveal different SQ% [10]. Furthermore, since a high SQ% obtained from tryptic peptides without missed cleavages indicates a more complete digest than the same high SQ% obtained from tryptic peptides with many missed cleavages, it is important to relate SQ% to the degree of missed cleavages of the peptides used to calculate this value [10]. It is reported that digestion efficiency can be determined by searching for the possible presence of intact protein in the total ion chromatogram [10]. We believe that a less complicated and demanding method for monitoring the efficiency and sufficiency of trypsin digestion that is independent of the mass spectrometry step will be more practical and easily implemented by many laboratories.

We realize that shotgun proteomics does not necessarily require complete digestion of proteins into peptides to be successful. However, for reproducibility and to be able to objectively compare the results of experiments within and between laboratories it will be necessary to standardize, in addition to the other parameters, the trypsin digestion step using an objective method. Obviously, the extent of digestion that will produce optimal protein identification and sequence coverage will have to be empirically determined for each sample type, when a new batch of enzyme is acquired or when a new or modified protocol is to be used. In this experiment, digestion of plasma and serum under the same conditions resulted in different extents of digestion and proteomics outcomes. This means that for improved efficiency, consistency and reproducibility the exact duration of digestion, the “sweet spot,” that produces the best amount of identified proteins and sequence coverage should be determined for each shotgun proteomics experiment.

To monitor protein digestion routinely, a fast and inexpensive method that requires a small amount of digested sample will be ideal. The Bioanalyzer has the advantage of being a sensitive, reproducible, and well accepted method for protein analysis. It provides an electrophoresis image that will show the presence or absence of protein peaks. However, the ProDM kit proved to be a faster and less expensive method for monitoring tryptic digestion compared to the Bioanalyzer. Specifically, in addition to the cost of the Bioanalyzer Reader which could be as high as $17,000, a Protein-80 chip for 1–10 samples was purchased for about $36, and a minimum of 60 min was required for one analysis. Conversely, ProDM only required an in-house bench top spectrophotometer, the cost per sample was less than $3.00, and less than 15 min was required for one analysis. A direct comparison of the ProDM and Bioanalyzer approach therefore shows that the Bioanalyzer approach requires about 20 *μ*L more digested sample, the cost of each assay was about 10 times more than that of ProDM, and the time required to complete the assay was at least 4 times more than the ProDM process. Hence ProDM, a colorimetric method could be a better, and an alternative method for screening tryptic digest to identify the extent of digestion that yields optimal proteomics results. ProDM could help take away the guess work and avoid time/sample wastage due to insufficient or excessive digestion.

## 4. Conclusion

Taken together, this study demonstrates the need to monitor and standardize the protein digestion step to improve (a) reproducibility, (b) protein identification efficiency, and (c) protein sequence coverage. This is particularly critical in proteomics applications like protein expression profiling, quantitative proteomics, and biomarker identification, where run-run reproducibility is vitally important and the more the number of proteins identified the better. We observed that the extent of protein digestion influenced the mass spectrometry results, indicating that the protein digestion step needs to be optimized to improve the success of proteomics experiments and prevent the wastage of valuable samples and time. Furthermore, the “extent” of protein(s) digested, rather than the “duration” of trypsin digestion, may be a more objective method for standardizing and comparing results, since the type of protein, complexity of the protein mixture, and specific activity of the enzyme will affect the total time required to achieve the extent of digestion that will yield the best proteomics outcome. ProDM, a simple colorimetric assay which allows the precise calculation of the percentage of proteins digested in samples, could help laboratories monitor protein digestion prior to mass spectrometry, identify digestion conditions that yield the best outcome, standardize the digestion step, and optimize protein identification and sequence coverage. It is expected that this paper will stimulate additional studies that will increase our understanding of the significance of monitoring and standardizing the protein digestion step.

## Figures and Tables

**Table 1:** Percentage of proteins digested calculated with ProDM, total number of unique peptides sequenced, and proteins identified by mass spectrometry in plasma and serum after tryptic digestion for 8 h and 24 h at 37°C.

Compared parameter	Plasma		Serum	
	8 h	24 h	8 h	24 h
% protein digested calculated with ProDM	46.5	56.1	46.2	50.2
No. of peptides sequenced by LC/MS/MS	991	733	897	693
Total no. of unique proteins identified	125	118	127	107

**Table 2:** Percent of peptides with 1+, 2+, and 3+ charge states in 8 h and 24 h digested plasma and serum samples.

Charge state	% of charged peptides in plasma		% of charged peptides in serum	
	8 h	24 h	8 h	24 h
1+	0.93	1.99	0.76	3.2
2+	72.43	68.16	85.50	82.4
3+	26.64	29.85	13.74	14.4
